# Is authentic leadership always good for employers? A perspective of time management

**DOI:** 10.3389/fpsyg.2022.892909

**Published:** 2022-11-16

**Authors:** Chih-Jen Lee, Stanley Y. B. Huang, Tai-Wei Chang, Shih-Chin Lee

**Affiliations:** ^1^Master Program of Financial Technology, Ming Chuan University, Taipei City, Taiwan; ^2^Graduate School of Resources Management and Decision Science, National Defense University, Taipei City, Taiwan; ^3^Department of Finance, Chihlee University of Technology, New Taipei City, Taiwan

**Keywords:** organizational inclusion, organizational embeddedness, social capital activities, time management, authentic leadership

## Introduction

Authentic leadership refers to leaders who use self-awareness, relationship transparency, internalized ethics, and balanced handling to guide employees (Walumbwa et al., 2008; Wen et al., 2021; Huang et al., 2022). However, an overly authentic environment can lead employees to optimize their limited resources to decide which activities to invest in because they don't have to worry about negative outcomes in a sincere environment, which past surveys have overlooked. In fact, past surveys have almost adopted a positive lens to investigate the impact of authentic leadership on positive employee behavior (Cao et al., 2020; Marques-Quinteiro et al., 2021) and the softening effect on negative employee behavior (Jang and Kim, 2021; Monzani et al., 2021), but these surveys have ignored the possible negative outcomes of authentic leadership. In response to these literature streams, the current research proposes a new stream that authentic leadership will reduce employees' investment in social capital activities through the mediating role of organizational embeddedness, and that relationship is moderated by organizational inclusion. In fact, from a time management perspective (Claessens et al., 2007), employees do deep calculations to reduce their investment in work because the likelihood of being fired in an authentic, ethical, and inclusive environment is low. That is, employees with high organizational embeddedness mean that these employees have high job security and low job mobility (Mitchell et al., 2001), and these employees no longer need to invest excessive resources to maintain their jobs, which may reduce investment in social capital activities. Social capital activities refer to those employees investing resources to develop relationships with external experts and internal colleagues to achieve career success (Coleman, 1990; Xie et al., 2021; Yu et al., 2021). Also, in an organization's highly inclusive environment, employees don't worry about negative outcomes because the organization tolerates employee behaviors. Organizational inclusion means senior leadership's commitment to promoting inclusion, employees' ability to influence organizational decisions, and fair/equitable treatment by management (Sabharwal, 2014).

Taken together, the current research plans to build a new stream of authentic leadership literature to explain why it can negatively affect investment in social capital activities through the mediating role of organizational embeddedness, and that relationship is moderated by organizational inclusion. The negative impact of authentic leadership is significant because it may worsen employee career development and organizational performance.

## Literature reviewing

**Figure 1:**
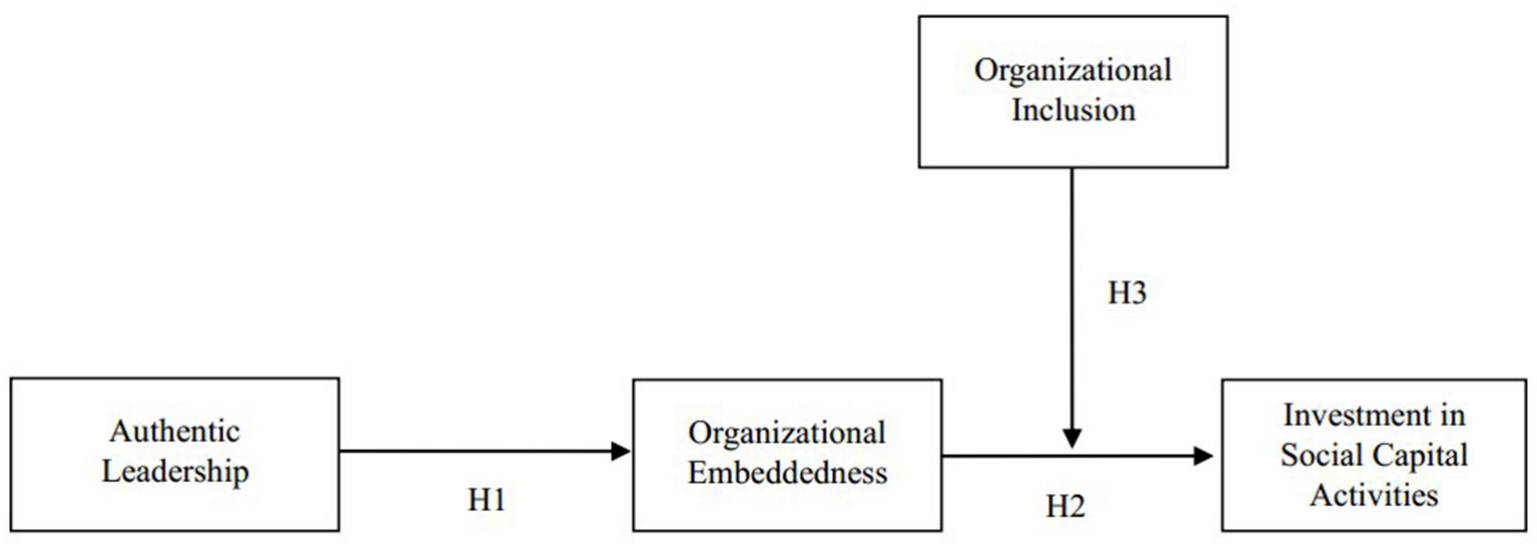
Research model of this research.

The current research constructs a novel moderated mediation model of organizational embeddedness to describe the effects of authentic leadership on organizational embeddedness and investment in social capital activities, and the relationship is moderated by organizational inclusion (Figure 1).

### Authentic leadership and organizational embeddedness

Organizational embeddedness represents an employee's decision to remain in the organization, and past researchers (Mitchell et al., 2001) proposed three dimensions of organizational embeddedness, including employee-organizational bond, fit, and commitment. The current research argues that authentic leadership increases the organizational embeddedness of employees. In fact, authentic leaders use relationship transparency, internalized ethical perspective, and a balanced approach to manage their followers, so the process of authentic leadership can shape an ethical environment that allows those followers to develop stronger bonds with their colleagues. Furthermore, leaders can communicate organizational values to subordinates (Huang et al., 2022), and leaders can guide subordinates' self-values to align with organizational values, which will increase fit and commitment between employees and their organizations.

Proposition 1: Authentic Leadership positively affects organizational embeddedness.

### Organizational embeddedness and investment in social capital activities

Employees with a high level of organizational embeddedness represent two things. The first is that these employees have a higher fit with the organization, so these employees are less likely to find new jobs, which can reduce the incentive for these employees to invest in social capital activities, such as building relationships with outside experts. The second is that these employees are highly connected to the organization, so the likelihood of these employees being fired should be low, which reduces the incentive for these employees to invest in social capital activities, such as building relationships with internal colleagues. In fact, these employees invest a lot of time and energy in maintaining relationships with external experts and internal colleagues, but their resources are limited. Based on a time management perspective (Claessens et al., 2007), these employees should account for these limited resources to reduce work resource allocation because the likelihood of being fired or finding a new job is low.

Proposition 2: Organizational embeddedness negatively affects investment in social capital Activities.

### The moderating role of organizational inclusion

As above-mentioned, employees may reduce their investment in jobs (e.g., social capital activities), because they no longer worry about losing their jobs in a status of high-level organizational embeddedness. In fact, organizational inclusion may boost the relationship between organizational embeddedness and investment in social capital activities, because employees can feel safer reducing more investment in social capital activities in a more inclusive environment. That is to say, employees in the status of a higher level of organizational inclusion may reduce more investment in social capital activities than employees in the status of a lower level of organizational inclusion.

As noted above, employees may invest less in their jobs (e.g., social capital activities) because they no longer fear losing their jobs in a state of high levels of organizational embeddedness. In fact, organizational inclusion may deteriorate the relationship between organizational embeddedness and investment in social capital activities, as employees can invest less in social capital activities in a more inclusive environment. That is, employees who perceive more organizationally inclusive are likely to reduce their investment in social capital activities more than those who perceive less organizationally inclusive.

Proposition 3: Organizational inclusion will negatively moderate the relationship between organizational embeddedness and investment in social capital activities.

## Discussion

The current research adopts a time management perspective (Claessens et al., 2007) to reveal the negative impact of authentic leadership on investment in social capital activities, and that relationship is moderated by organizational inclusion. In fact, previous surveys have always examined the positive impact of authentic leadership on employee behaviors, but few studies have demonstrated the negative impact on employee behaviors. Thus, the current study opens up a new milestone in authentic leadership.

Based on the perspective of time management (Claessens et al., 2007), the current research draws on the literature on leadership, organizational embeddedness, and organizational inclusion to initiate an investment mechanism for social capital activities, which has interdisciplinary contributions.

Building on the research model of the current research, the current research shows that there are multiple management strategies that firms can employ to mitigate the negative impact of authentic leadership on investment in social capital activities, such as job rotation, as it reduces the negative impact of organizational embeddedness.

Finally, it is possible that other activities may also strive staff to rationalize their working time, so future research should deeply scrutiny other key activities. For example, human capital activities may be one of the other activities that may also strive staff rationalize their working time, because human capital activities also consume staff many resources to realize knowledge skills (Wang et al., 2019; Hu and Yao, 2021; Shen et al., 2021), such as the education training and obtaining an advanced degree.

## Author contributions

C-JL: conceptualization and writing—original draft. SH: project administration, supervision, and writing—revised draft. T-WC and S-CL: literature collection, idea generation, and writing, review and editing—revised draft. All authors contributed to the article and approved the submitted version.

## Conflict of interest

The authors declare that the research was conducted in the absence of any commercial or financial relationships that could be construed as a potential conflict of interest.

## Publisher's note

All claims expressed in this article are solely those of the authors and do not necessarily represent those of their affiliated organizations, or those of the publisher, the editors and the reviewers. Any product that may be evaluated in this article, or claim that may be made by its manufacturer, is not guaranteed or endorsed by the publisher.
